# Multi-residue pesticides analysis in water samples using reverse phase high performance liquid chromatography (RP-HPLC)

**DOI:** 10.1016/j.mex.2018.07.005

**Published:** 2018-07-09

**Authors:** Sneh Rajput, Arpna Kumari, Saroj Arora, Rajinder Kaur

**Affiliations:** Department of Botanical & Environmental Sciences, Guru Nanak Dev University, Amritsar, Punjab, India

**Keywords:** Quantitative analysis, Multi-residue pesticides, RP-HPLC, Pond water samples

## Abstract

India is one of the leading suppliers of agrochemicals and has the largest pesticide industry in Asia. Among various Indian states, Punjab is the primary user of pesticides. Presence of pesticide residue in water and food products of Punjab is well documented. The present study was designed to envisage the level of pesticide contamination in pond water of eleven villages of Amritsar district of Punjab, India. A rapid and concurrent method for the identification and quantification of pesticides in water samples was developed and validated. The method validation parameters exhibited high sensitivity of the developed method and the proficiency for the identification and quantification of pesticide residues in water samples. The RP-HPLC method described here

• is a novel method which is applicable for simple, rapid and precise detection of pesticides.
• 40.02% of water samples were found contaminated with multi-residue pesticides.
• carbofuran was the most abundant pesticide which was present in 18.18% samples.

## Graphical abstract

**Figure:**
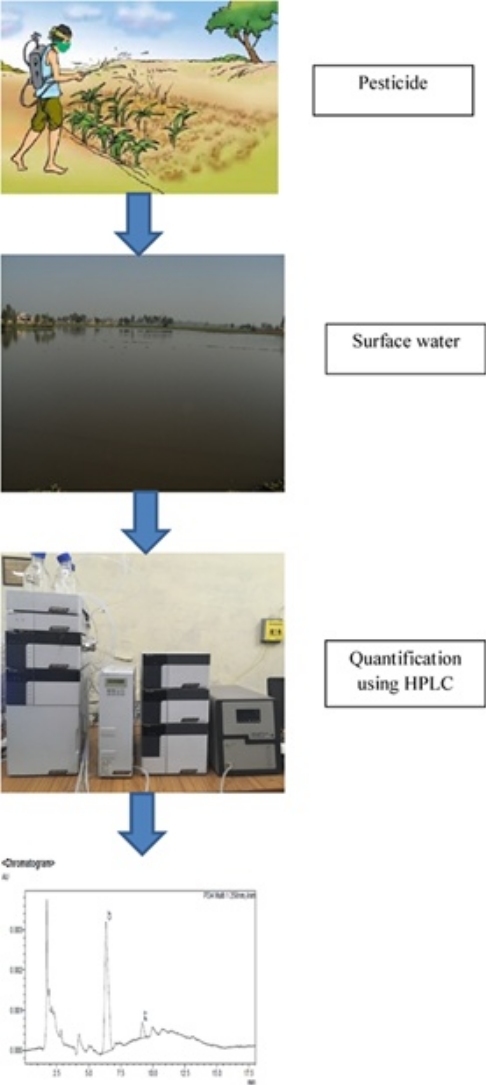

## Method details

### Sample collection and sampling sites

**Fig. 1:**
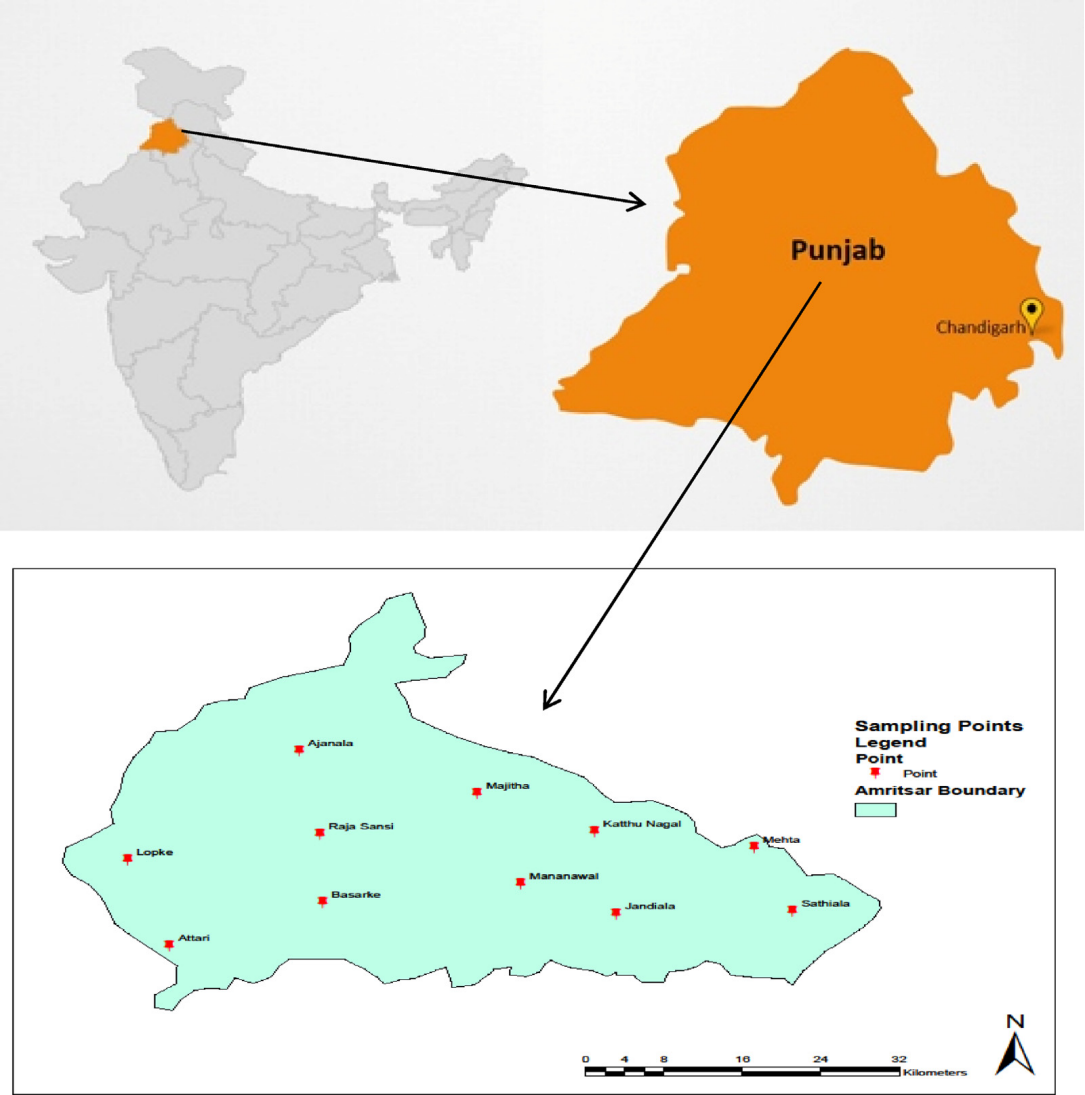
Study area and monitoring sites.

Punjab is mainly divided into three regions namely Malwa, Doaba and Majha. Amritsar is one of the four districts located in the Majha region and the second most populated district of Punjab. The weather of Amritsar shows extreme variations throughout the year. It becomes extremely hot during summers and extremely cold during winters. On an average, July is the drizzliest month and October is the driest. For the present study, the map of Amritsar district of Punjab (India) was prepared and gridding was done accordingly for the systematic collection of samples (Fig. 1). Water samples were collected from eleven different ponds during monsoon, post monsoon, summer and winter season for two consecutive years (July 2015–May 2017) using global positioning system. Sampling sites along with their coordinates are given in Table 1.

**Table 1:** Sampling sites along with their coordinates.

S. No.	Name	Site code	Coordinates	
			Latitude	Longitude
I	Baserke Gallan	BG	31°61′77″ N	74°71′90″ E
II	Ajnala	AJ	31°84′00″ N	74°76′00″ E
III	Raja Sansi	RS	31°72′45″ N	74°78′60″ E
IV	Manawala	MW	31°74′06″ N	74°68′83″ E
V	Majitha	MJ	31°76′00″ N	74°95′00″ E
VI	Lopoke	LO	31°71′70″ N	74°63′27″ E
VII	Attari	AT	31°69′31″ N	74°65′79″ E
VIII	Jandiala	JA	31°58′93″ N	75°05′68″ E
IX	Sathiala	SA	31°55′50″ N	75°26′55″ E
X	Mehta	ME	31°63′39″ N	74°87′22″ E
XI	Kathunangal	KN	31°73′24″ N	75°02′31″ E

### Chemicals

In the selection of the pesticides to be analysed, priority was given to the pesticides commonly used in Punjab. HPLC acetonitrile (CAS: 75-05-8) and HPLC water were used as mobile phase and were procured from Himedia Laboratories Private Limited (Mumbai). All other reagents were of analytical grade and were procured from Sigma Aldrich. List of pesticides along with their properties is given in Table 2.

**Table 2:** List of pesticides used for quantification.

Pesticide	Chemical formula	Use	CAS number	Purity
Aldicarb	C_7_H_14_N_2_O_2_S	Multi-use pesticide	116-06-3	99.9%
Atrazine	C_8_H_14_ClN_5_	Herbicide	1912-24-9	98.8%
Carbofuran	C_12_H_15_NO_3_	Broad spectrum insecticide	1563-66-2	99.9%
Carbendazim	C_9_H_9_N_3_O_2_	Fungicide	613-048-00-8	99.2%
Methoxychlor	C_16_H_15_Cl_3_O_2_	Insecticide	72-43-5	98.7%
Parathion methyl	C_8_H_10_NO_5_PS	Insecticide as well as acaricide	298-00-0	99.7%
Spiromesifen	C_23_H_30_O_4_	Insecticide	283594-90-1	98.8%

### Extraction procedure

2 litres of water sample was extracted with 100 mL of n-hexane by shaking several times in a separating funnel. The extraction procedure was repeated thrice. The organic layers were collected and passed through anhydrous sodium sulphate to remove water content. The collected fraction was reduced to 1 mL on rotary evaporator. 2 mL of acetonitrile was then added and samples were filtered through syringe filtered of 0.22 μm prior to analysis.

### Chromatographic conditions

The analysis of pesticides was carried out using 130 MPa Shimadzu RP-HPLC-PDA system (Nexera) purchased from Shimadzu Corporation (Japan). The system was coupled with degasser, solvent mixing unit, autosampler, column oven, PDA detector and system controller (CBM-20 A). The chromatographic separation was carried out using C_18_ column with dimensions of 250 × 4.6 mm and pore size of 5 μm. The mobile phase consisted of acetonitrile (A) and HPLC water (B). The mobile phase was filtered using 0.22 μm membrane and degassed for 30 min by sonication. The gradient elution started with 45% A and 55% B, continued with 30% B at 5.00 min., 20% B at 7.50 min., then 25% B at 9.00 min., followed by 35% B at 14.01 min. and stopped at 18.01 min. The flow rate was maintained at 1.00 mL/min, 12 μL injection volume, 38 °C column temperature and the maximum pressure limit of 15,000 psi. The operating conditions of HPLC are given in Table 3.

**Table 3:** Operating conditions of HPLC for method development.

Analytes	Aldicarb; Atrazine; Carbofuran; Carbendazim; Methoxychlor; Parathion methyl; Spiromesifen
Gradient elution	Solvent B: 0.01 min. 55% B; 5.00 min. 30% B; 7.50 min. 20 % B; 9.00 min. 25% B; 14.01 min. 35% B; 18.01 min. Stop
Total run time	18.01 min.
Column	C_18_ column
Column Temperature	38 °C
Flow rate	1 mL/min
Injection volume	12 μL
Detection wavelength	258 nm

Where A is Acetonitrile; B is water.

### Preparation of solution and mobile phase

The stock solution (500 mg/L) of each pesticide was prepared in acetonitrile and working solution was prepared through serial dilution of stock.

### Method development and validation indices

Method validation indices were performed according to International Conference on Harmonisation of Technical Requirement for Registration of Pharmaceutical for Human Use (ICH, 2005).

### Standard curve

Calibration curve was obtained using peak area versus concentration of each analyte. Subsequently, these standard curves were used for quantification of pesticide residues in water samples.

### Precision

The precision of system was observed to access the repeatability which is expressed as percentage of relative standard deviation (% RSD). % RSD was calculated from six replicates of individual pesticide.

% RSD = (Standard deviation/Mean peak area) × 100

### Accuracy

Accuracy of analytical procedures is also termed as trueness. It is the proximity between the conventional value and the observed value. The accuracy is expressed as % recovery which was calculated using the equation as follows:

% Recovery = (Detected concentration/Nominal concentration) × 100

### LOD and LOQ

Limit of detection (LOD) and limit of quantification (LOQ) of the RP-HPLC-PDA method for all seven pesticides was observed. LOD and LOQ were calculated on the basis of signal-to-noise ratio (S/N) which is 3.3 and 10 respectively according to ICH guidelines, 2005 [1].

### Method validation and quantification of pesticide residues

All the water samples collected from different sampling sites during different seasons were quantified for the presence of pesticides residue using RP-HPLC. The present method exhibited high linearity range for all pesticides with the maximum positive correlation coefficient (*r*) of 0.99 which contributed towards the good correlation between peak area and concentrations (Table 4). The present study revealed that different pesticides and their concentration varied significantly during different seasons (Table 5). Maximum number of pesticides were observed during monsoon season (2015) followed by winter season (2016) and post monsoon season (2016). No pesticide was detected in water samples collected during the summer season (2016). Among the various sampling sites, Mehta was the most pesticides contaminated site followed by Jandiala, Ajnala and Kathunangal sites whereas Attari was the least contaminated site.

**Table 4:** Method development and validation.

Analyte	Linearity range (mg/L)	%Recovery	%RSD	LOD	LOQ	Regression equation	Correlation coefficient (r)
AC	2.5–500	102.45 ± 3.22	0.36	0.70	2.13	Y = 1986.2x-12177	0.99
AZ	2.5–500	97.91 ± 2.49	0.08	0.33	1.00	Y = 4426.4x-10850	0.99
CF	2.5–500	105.76 ± 5.08	0.31	1.20	3.62	Y = 1046.6x-10200	0.99
CD	2.5–500	103.48 ± 3.71	0.80	0.22	0.66	Y = 1180.6x-2940.6	0.99
MC	2.5–500	105.72 ± 5.08	0.98	0.97	2.95	Y = 14419x+158534	0.99
PT	2.5–500	100.66 ± 4.23	0.52	0.18	0.53	Y = 7684.4x-67036	0.99
SF	2.5–500	103.11 ± 4.29	0.83	2.41	7.30	Y = 140.6x+2045.5	0.99

**Table 5:** Multi residue detection of pesticides (mg/L) in water samples collected from different sites from July 2015–May 2017.

	Site	Season	AC	AT	CD	CF	PT
First year of sampling (July 2015-May 2016)	AJ	Monsoon	ND	ND	ND	**19.88**	**182.78**
	LO	Monsoon	**29.31**	**21.86**	ND	**21.48**	**33.24**
	SA	Monsoon	ND	4.57	ND	**171.39**	ND
	ME	Monsoon	ND	ND	ND	**18.86**	ND
	AJ	Post monsoon	ND	**2.53**	ND	ND	ND
	JA	Post monsoon	ND	**3.80**	ND	ND	ND
	ME	Post monsoon	ND	**4.52**	ND	**57.21**	ND
	AT	Winter	ND	ND	ND	**63.96**	ND
	SA	Winter	ND	ND	ND	**76.02**	ND
	ME	Winter	ND	ND	ND	ND	**187.2**

Second year of sampling (July 2016-May 2017)	AJ	Monsoon	ND	ND	ND	**74.14**	ND
	ME	Monsoon	ND	ND	ND	**84.03**	**88.79**
	JA	Monsoon	**10.49**	ND	ND	**48.73**	ND
	MW	Post monsoon	ND	ND	ND	**19.72**	ND
	SA	Post monsoon	ND	ND	ND	**19.82**	ND
	ME	Post monsoon	ND	**3.22**	**2.50**	**68.01**	ND
	JA	Post monsoon	ND	ND	ND	**19.66**	ND
	MJ	Winter	ND	**9.19**	**59.8**	ND	**18.16**
	BG	Winter	ND	ND	ND	**67.51**	**94.30**
	JA	Winter	ND	ND	**5.44**	ND	**177.84**
	ME	Winter	ND	ND	**8.3**	ND	ND
	MW	Summer	ND	ND	ND	ND	**177.74**
	AJ	Summer	ND	**5.66**	ND	ND	ND
	KN	Summer	**13.40**	ND	ND	**19.76**	ND

*ND- Not detected.

**Fig. 2:**
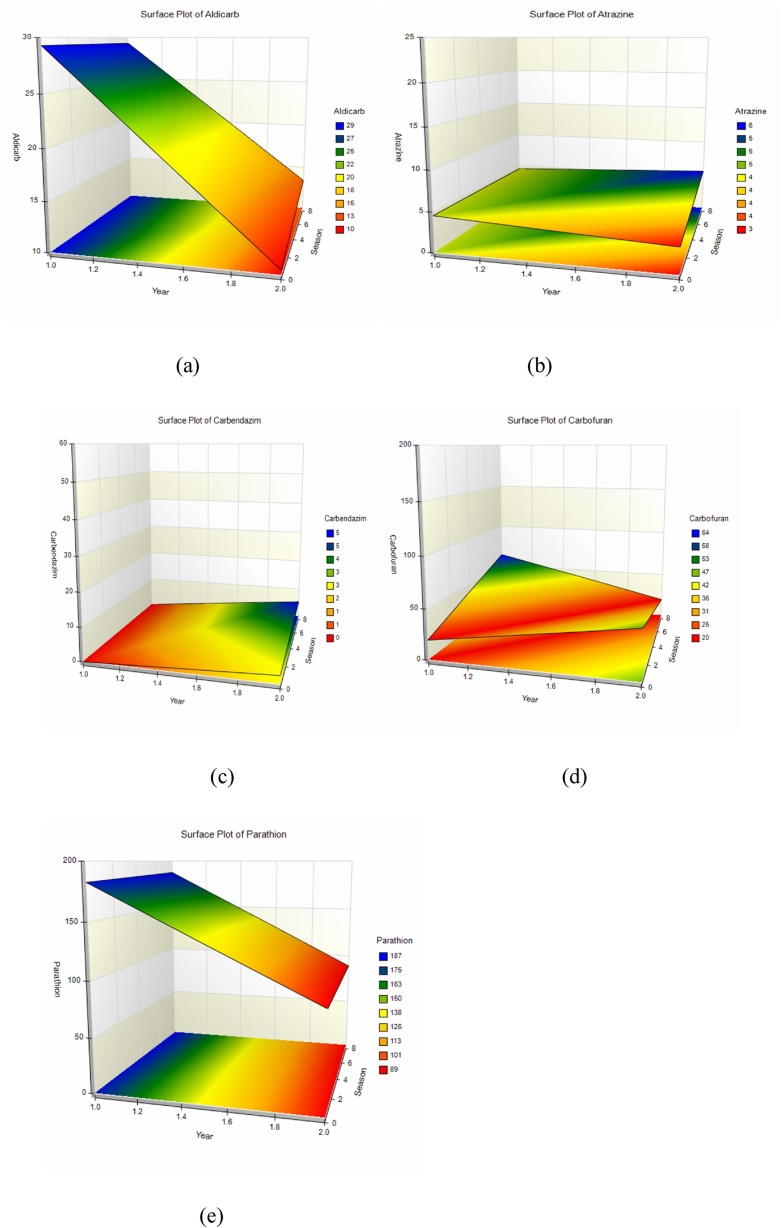
Three dimensional plots of pesticides as a function of season and time (a) Aldicarb (b) Atrazine (c) Carbendazim (d) Carbofuran (e) Parathion methyl.

The order of magnitude of pesticides in water samples was Carbofuran > Parathion methyl > Atrazine > Carbendazim > Aldicarb > Methoxychlor = Spiromesifen. Fig. 2(a–e) shows three dimensional plots of pesticides as a function of season and time.

## Additional information

In developing countries including India, about 800,000 people have lost their lives due to pesticides contamination since the beginning of Green revolution [2]. About 20,000 people lose their lives each year due to consumption of pesticides contaminated food and water [3]. According to a research conducted by The India pesticides industry analysis, the market size of pesticide industry in India will be 229,800 million by 2018. The use of pesticides in India has increased by 17% since 1955.

Pesticides are highly toxic, persistent and bio-accumulative in nature [4,5]. Due to their lipophilic characteristics, they get biomagnified in the food chain and may cause serious health consequences [6,7]. Health effects of pesticides include neurological disorder, leukaemia, reproductive damage, increased risk of Parkinson Disease, Alzheimer and birth defects [8,9]. Short-term exposure includes nausea, vomiting, dizziness, headache, skin rashes, muscle pain, respiratory disorders etc. [10]. Pesticides also get accumulated in human adipose tissue, blood and breast milk [[11], [12], [13]].

Varieties of pesticides are released into the environment every year which contaminate the aquatic ecosystem. This has become a serious environmental issue which is deteriorating the health of fresh as well as marine ecosystem. Pesticides in surface water can enter through agricultural runoff and pose a serious threat as they can undergo the transformations in water and can form more toxic compounds. There are various chromatographic techniques available for detection of pesticides. HPLC is one of the most reliable, economical and efficient method for the determination of pesticides in water, soil and food. HPLC is often combined with UV detector which is useful for the detection of a range of pesticides. The advantage of this method over other methods is that column can tolerate high pressure and flow rates.

### Correlation analysis

Table 6 shows the correlation analysis of detected pesticides where the significant correlation is marked bold. A strong positive correlation was found between aldicarb-atrazine (r = 0.790) and parathion methyl-atrazine (r = 0.694) at 0.05 level of significance, whereas a strong positive was found between aldicarb-parathion methyl (r = 0.522) at 0.01 level of significance. The significant correlation between pesticides indicates that their source of origin is similar, specifically from agricultural activities and surface runoff.

**Table 6:** Correlation matrix of pesticides (Pearson correlation coefficient).

Correlations					
	Aldicarb	Atrazine	Carbendazim	Carbofuran	Parathion methyl
Aldicarb	1				
Atrazine	0.790**	1			
Carbendazim	0.283	0.075	1		
Carbofuran	0.034	−0.136	−0.205	1	
Parathion methyl	0.522*	0.694**	−0.039	0.415	1

* Correlation significant at the 0.05 level.

** Correlation significant at the 0.01 level.

## Conclusion

A novel method was developed and validated for the quantification of multi-residue pesticides in water samples. The newly developed RP-HPLC method is applicable for simple, rapid and precise detection of pesticides residues at 38 °C column oven temperature with acetonitrile and water as mobile phase. The method has been proved to be effective for detection of multi-residue pesticides.

## Conflict of interest

The authors declare there is no conflict of interest.
